# Supplementary material for: Autophagy differentially regulates TNF receptor Fn14 by distinct mammalian Atg8 proteins
Source: Nat Commun. 2018 Sep 14;9:3744. doi: 10.1038/s41467-018-06275-1 (PMC6138730; doi:10.1038/s41467-018-06275-1)

“Autophagy differentially regulates TNF receptor Fn14 by distinct mammalian Atg8 proteins”

Winer et al.

Supplementary Information

Supplementary Figure 1

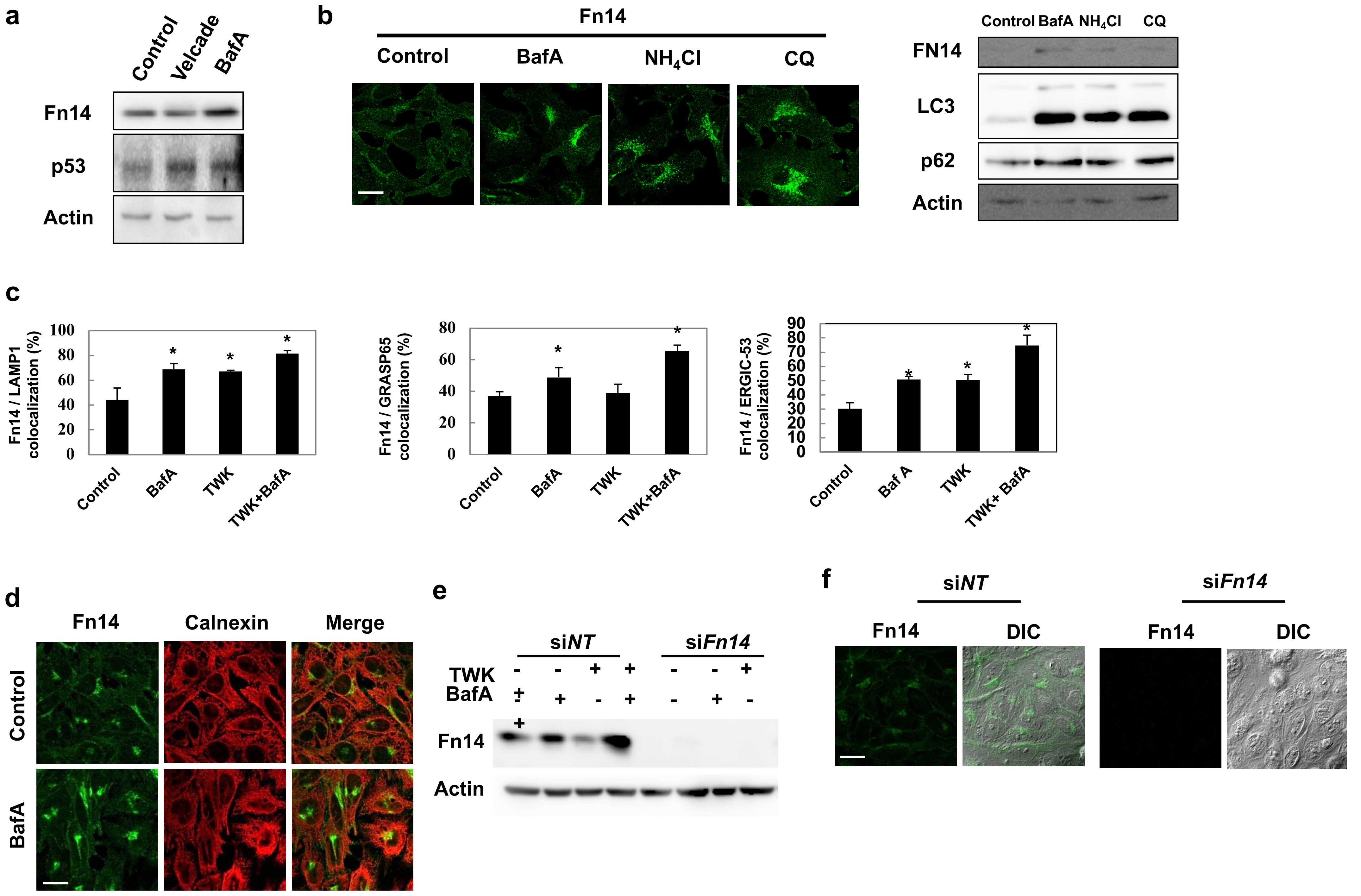

(a) HeLa cells were treated for 4h with BafA and Velcade as indicated and extracted proteins were immunoblotted. (b) HeLa cells were treated 16h with BafA, NH_4Cl or chloroquine (CQ) as indicated, followed by either immunostaining for Fn14 and analysis by confocal microscopy (scale bar, 20µm) (left panel) or protein extraction and immunoblotting (right panel). (c) Corresponding to Fig. 1b-d, colocalization of Fn14 with LAMP1, GRASP65 or ERGIC-53 n=5 fields (*, P < 0.05, comparisons by T test; mean ± s.e.m.). (d) HeLa cells were immunostained for Fn14 and Calnexin and analyzed by confocal microscopy (scale bar, 20µm). (e-f) HeLa cells were transfected si*NT* or siRNA against Fn14 (si*Fn14*), treated with TWK and BafA as indicated and extracted proteins were immunoblotted (e) or cells were immunostained for Fn14 and analyzed by confocal microscopy (scale bar, 20mm) (f).

Supplementary Figure 2

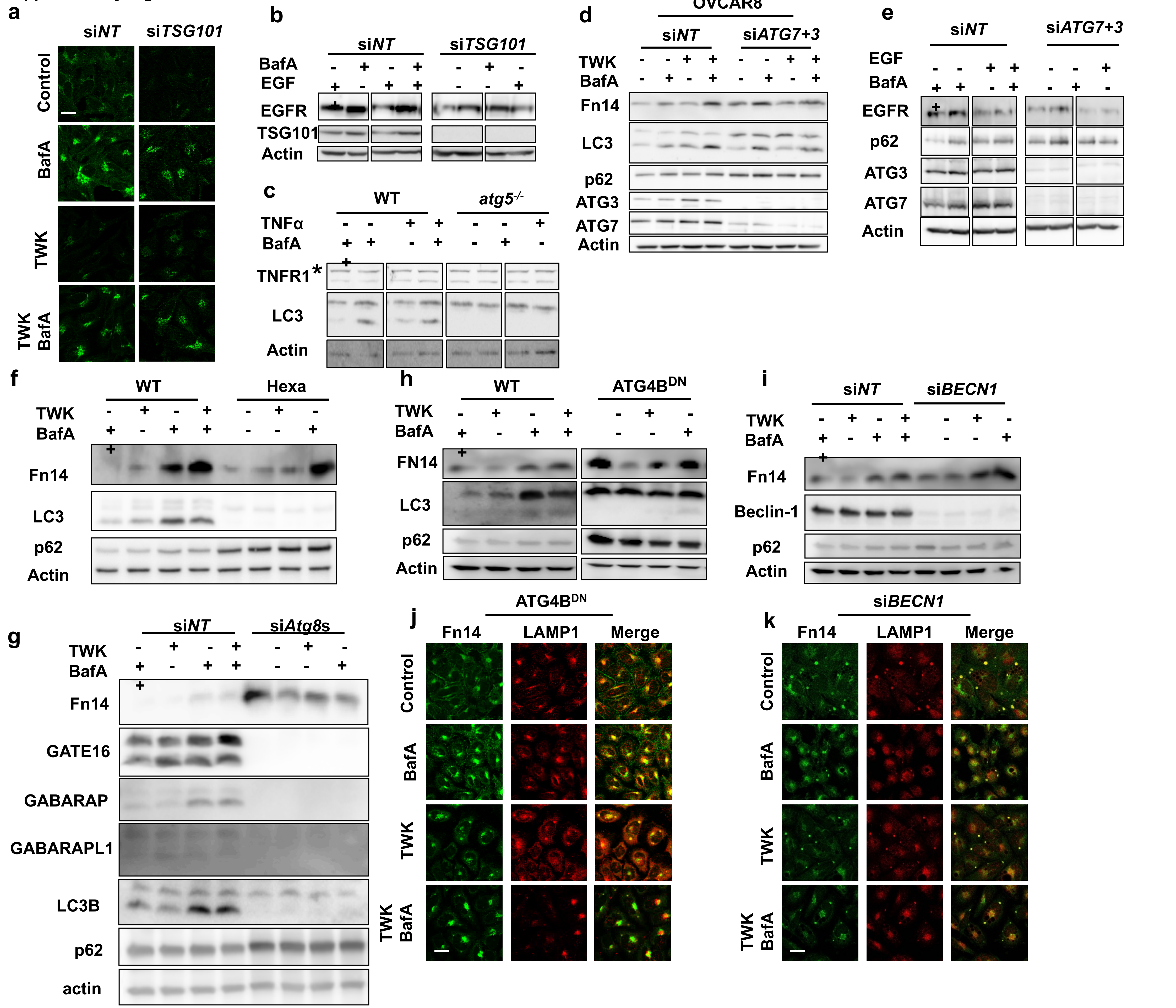

(a) HeLa cells transfected with si*NT* or si*TSG101* and treated with TWK and BafA as indicated were immunostained for Fn14 and analyzed by confocal microscopy (scale bar, 20mm). (b) HeLa cells transfected as in (a) were treated with BafA as indicated and then for 15min with EGF as indicated, and extracted proteins were immunoblotted. (c) WT or *atg5*$^{-/-}$ MEFs were transfected with expression plasmid of TNFR1, treated for 4h with TNFa and BafA as indicated and extracted proteins were immunoblotted. (d) OVCAR8 cells were transfected with si*NT* or si*ATG7+3*, treated with TWK and BafA as indicated and extracted proteins were immunoblotted. (e) HeLa cells were transfected as in (d), treated 4h with BafA and for additional 15min with EGF as indicated, and extracted proteins were immunoblotted. (f) HeLa WT cells or CRISPR knockout of all Atg8s (Hexa) were treated with BafA and TWK as indicated and extracted proteins were immunoblotted. (g-i) Cells were treated as in Fig. 2d (g), 2e (h) or 2f (i) and extracted proteins were immunoblotted. (j-k) Cells were treated as in Fig. 2e (j) or 2f (k), immunostained for Fn14 and LAMP1 and analyzed by confocal microscopy (scale bar, 20μm).

Supplementary Figure 3

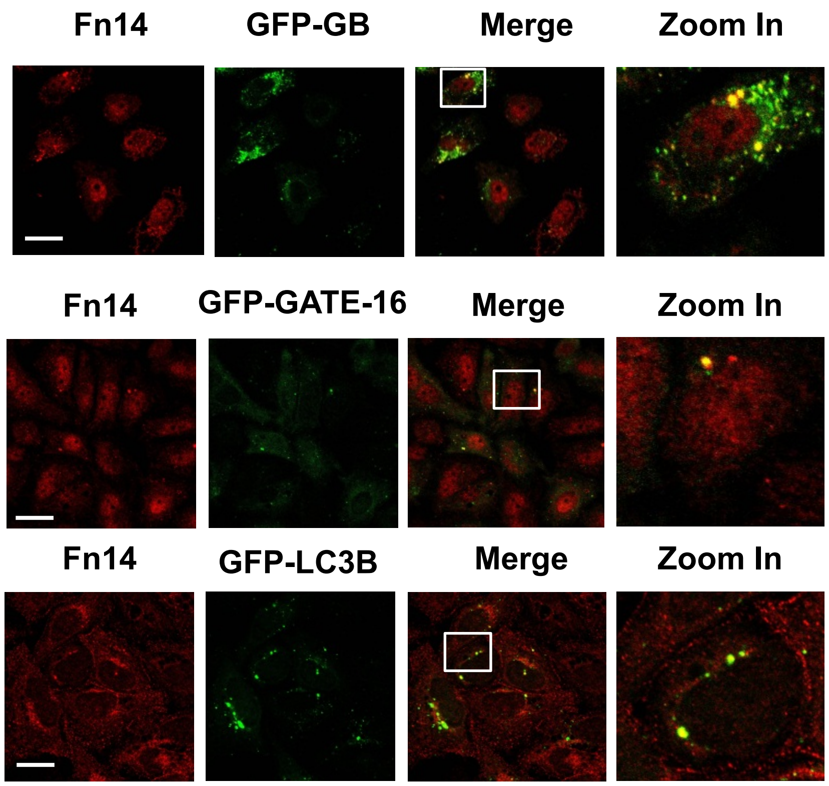

HeLa cells stably expressing GFP-GABARAP (GFP-GB), GFP-GATE-16 or GFP-LC3B were immunostained for Fn14 and analyzed by confocal microscopy (scale bar, 20μm). Large magnification of stained cells is presented in the right column.

Supplementary Figure 4

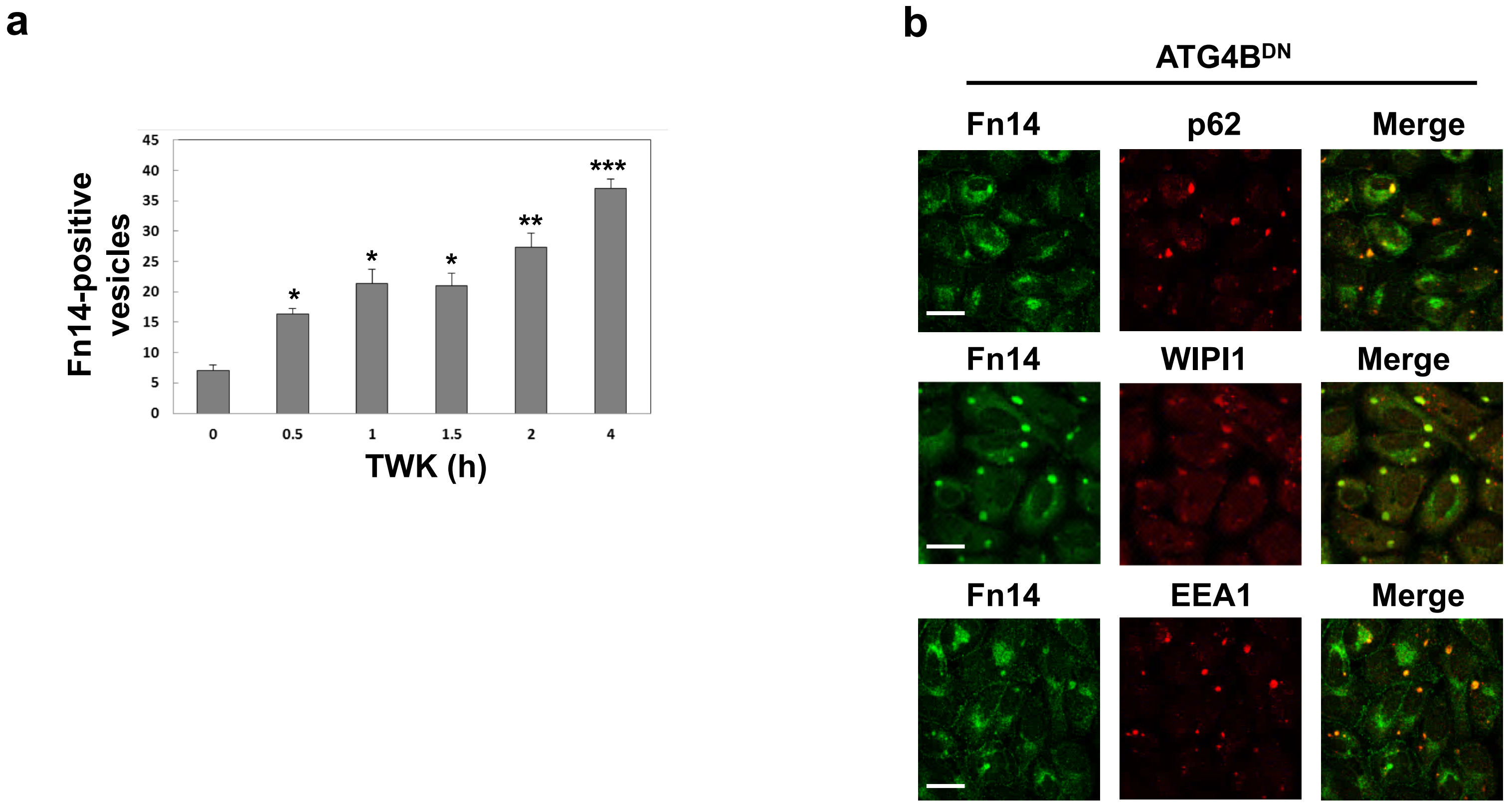

(a) Quantification of Fn14-positive vesicles in ATG4B^{DN} cells in Fig. 4a n=3 fields (*, $P < 0.05$; ** $P < 0.001$; ***, $P < 0.0001$, comparisons by ANOVA, mean ± s.e.m.) . (b) ATG4B^{DN} stable expression HeLa cells were starved 12h in EBSS, immunostained for Fn14 and p62, WIPI1 or EEA1 and analyzed by confocal microscopy (scale bar, 20μm).

Supplementary Figure 5

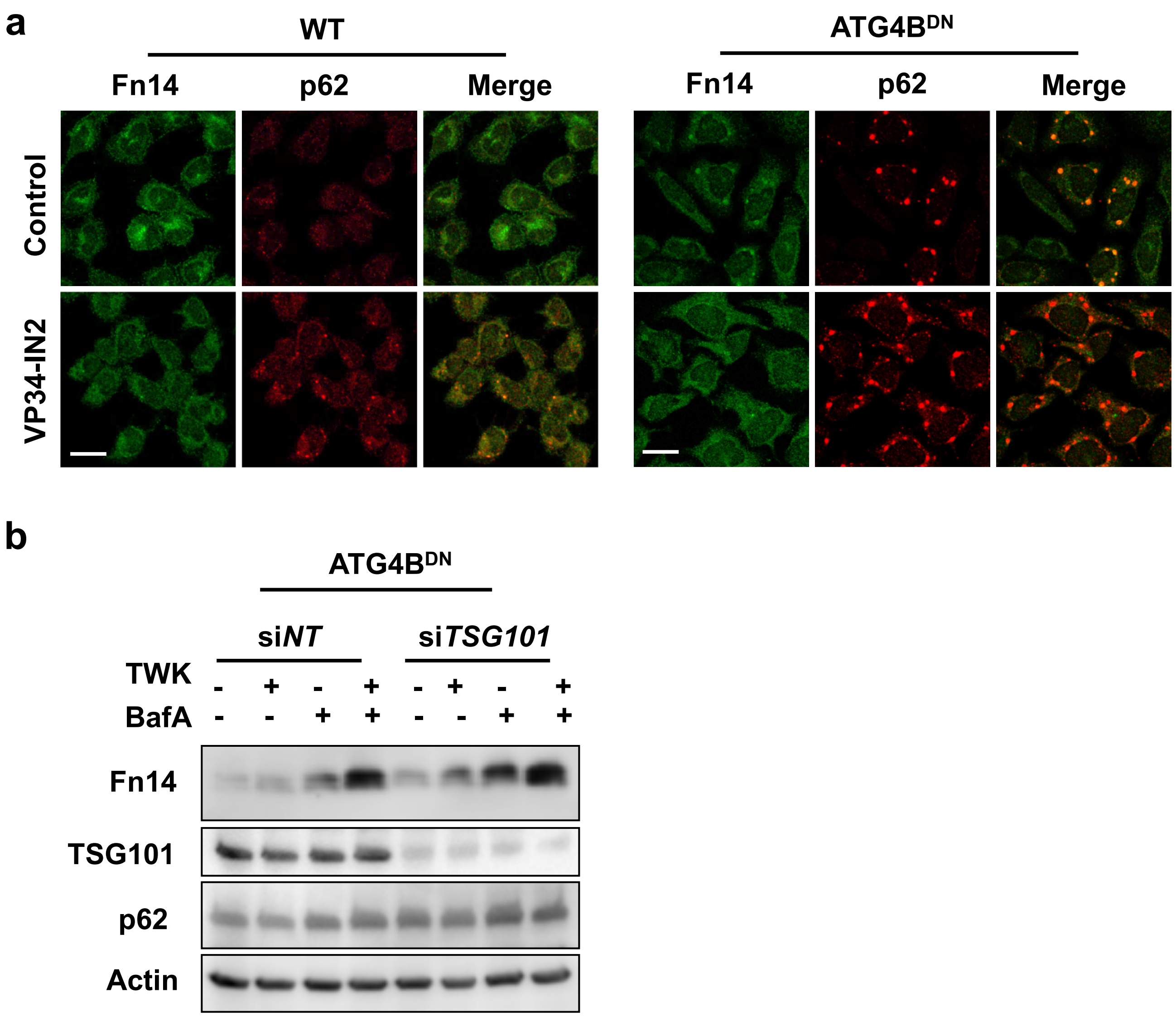

(a) WT or ATG4B^{DN} stable expression HeLa cells were treated for 4h with VPS34-IN2 as indicated, immunostained for Fn14 and p62 and analyzed by confocal microscopy (scale bar, 20μm). (b) Cells were treated as in Fig. 5d and extracted proteins were immunoblotted.

Supplementary Figure 6

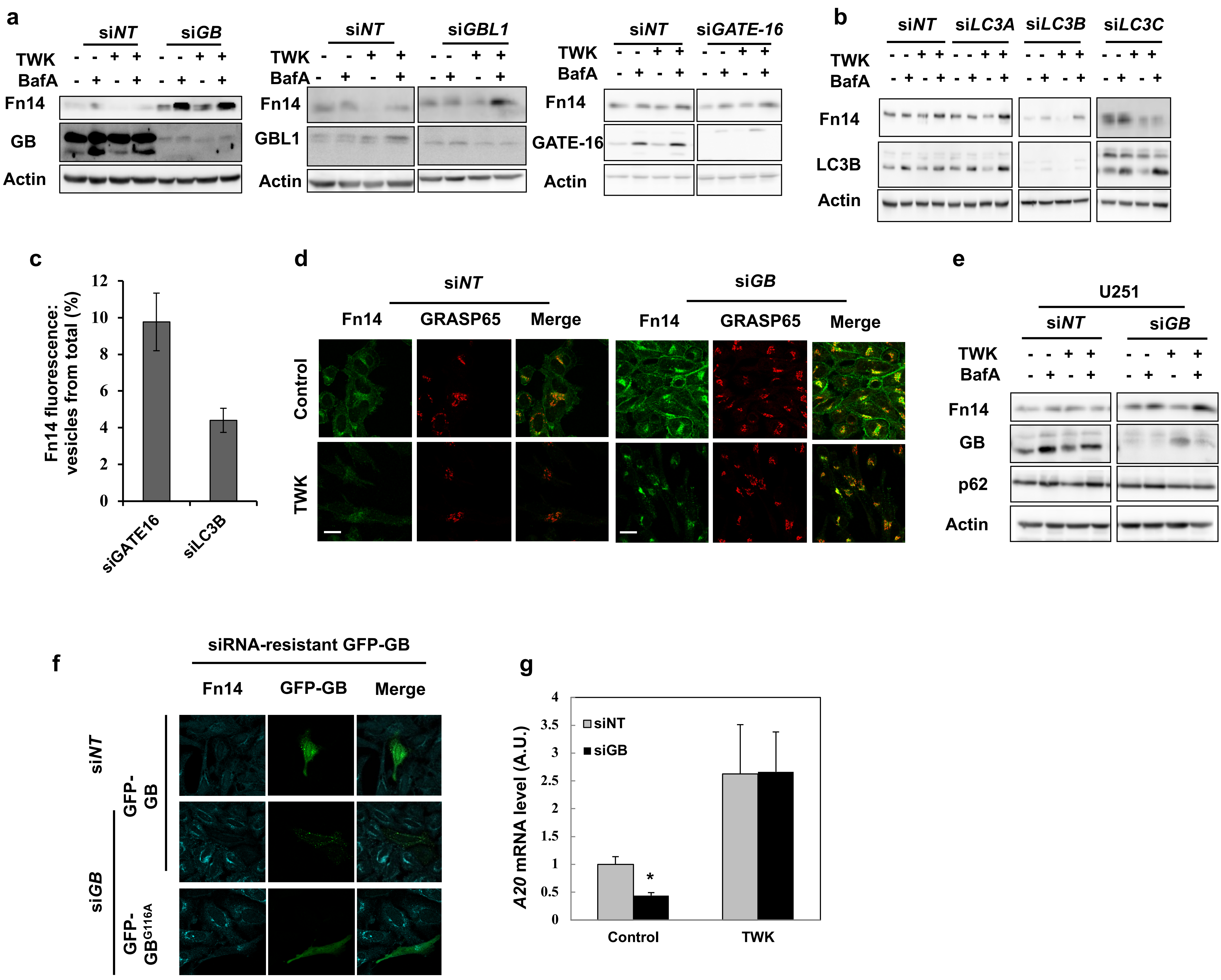

(a) HeLa cells were transfected with si*NT*, si*GB*, si*GBL1* or si*GATE-16*, treated with TWK as indicated and extracted proteins were immunoblotted. (b) HeLa cells transfected with si*NT*, and si*LC3A*, si*LC3B* or si*LC3C* were treated with BafA and TWK as indicated and extracted proteins were immunoblotted. (c) Fn14 fluorescence in vesicles *versus* total in Fig. 6a (for siGATE-16 n=15 cells, for siLC3B n=13, mean ± s.d. (d) HeLa cells transfected with si*NT* or si*GB* were treated with TWK as indicated, immunostained for Fn14 and GRASP65 and analyzed by confocal microscopy (scale bar, 20μm). (e) U251 cells transfected with si*NT* or si*GB* were treated with TWK and BafA as indicated and extracted proteins were immunoblotted. (f) Cells were transfected with siRNA as in (d) and after 24h were transfected with overexpression plasmid of siRNA-resistant GFP-tagged GABARAP – WT (GFP-GB) or non-lipidated mutant (GFP-GB^{G116A}) – for further 48h. Cells were then immunostained for Fn14 and analyzed by confocal microscopy (scale bar, 20 μm). (g) Cells transfected with siRNA as in (d) were lysed and subjected to RT-PCR analysis of A20 mRNA as described under Materials and Methods (*, $P < 0.05$, comparisons by T test; mean ± s.e.m. n=3 biological repeats).

Supplementary Figure 7

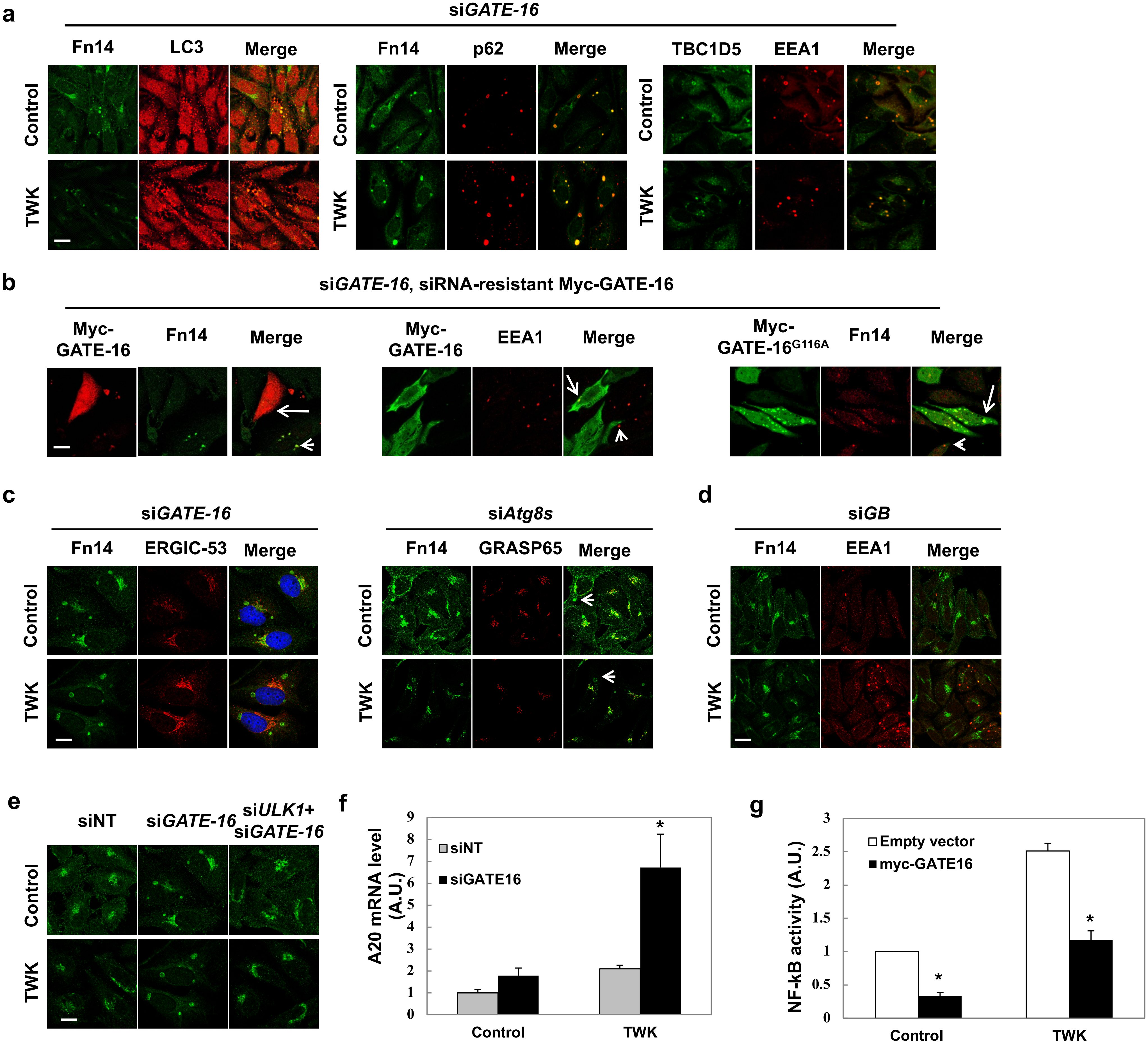

(a-e) HeLa cells transfected siRNA as follows were treated with TWK as indicated, immunostained as follows and analyzed by confocal microscopy (scale bar, 20 µm): (a) si*GATE-16*, stained for Fn14 and LC3B or p62, or for TBC1D5 and EEA1; (b) si*GATE-16* for 24h, and then transfected with overexpression plasmid of siRNA-resistant Myc-tagged GATE-16 – WT (Myc-GATE-16) or non-lipidated mutant (Myc-GATE-16^{G116A}) – for further 48h prior to TWK, stained for Myc and Fn14 or EEA1; (c) si*GATE-16* or si*Atg8s*, stained for Fn14 and ERGIC-53 or GRASP65, respectively; (d) si*GB*, stained for Fn14 and EEA1; (e) si*NT* si*GATE-16* or si*GATE-16* and si*ULK1*, stained for Fn14. (f) Cells transfected and treated as in (a) were lysed and subjected to RT-PCR analysis of A20 mRNA as described under Materials and methods (*, $P < 0.05$, comparisons by T test; mean ± s.e.m. n=4 biological repeats). (g) HeLa cells were transfected with si*GATE-16* and after 24h were transfected with luciferase expression constructs as detailed under Materials and Methods, treated with TWK and further subjected to luciferase assay as detailed to assess NF-kB activity (*, $P < 0.05$, comparisons by T test; mean ± s.e.m. n= 3 biological repeats).

Supplementary Fig. 8

Uncropped data for Fig. 1a

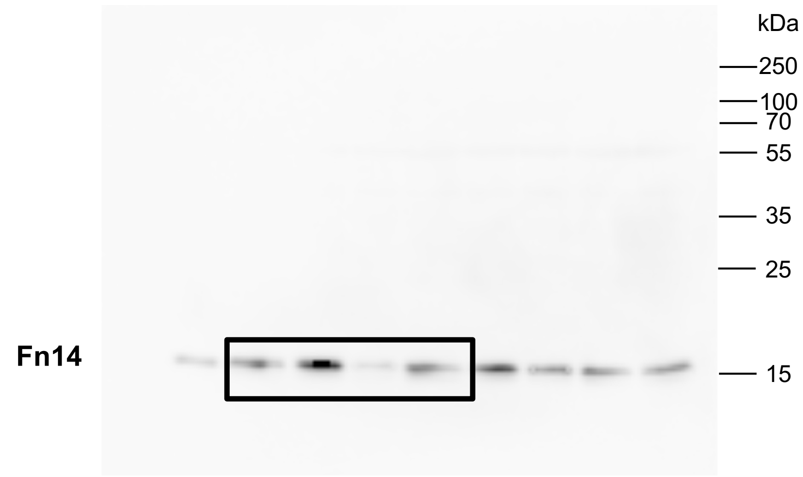

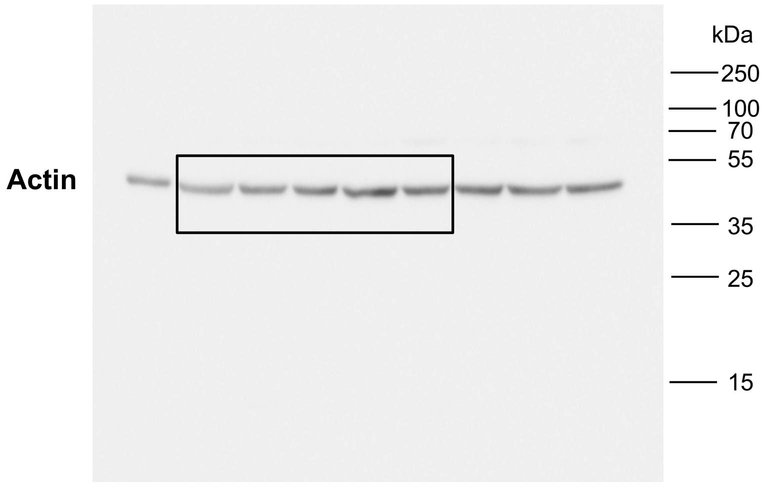

Supplementary Fig. 9

Uncropped data for Fig. 2a

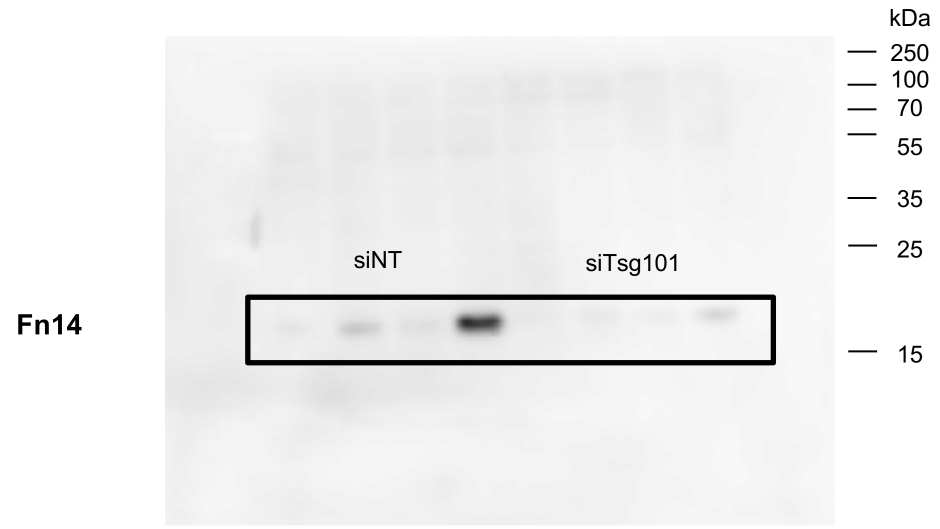

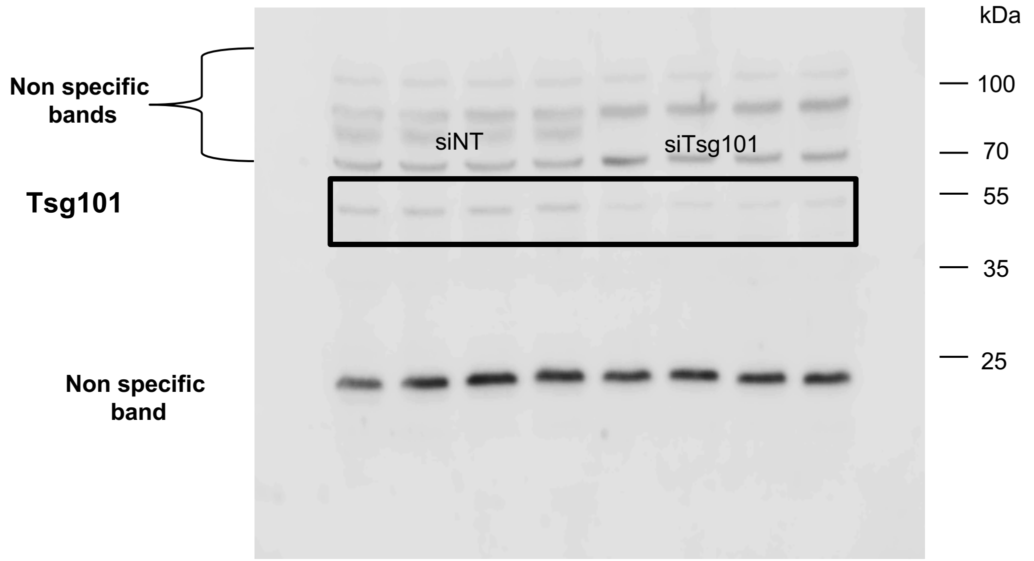

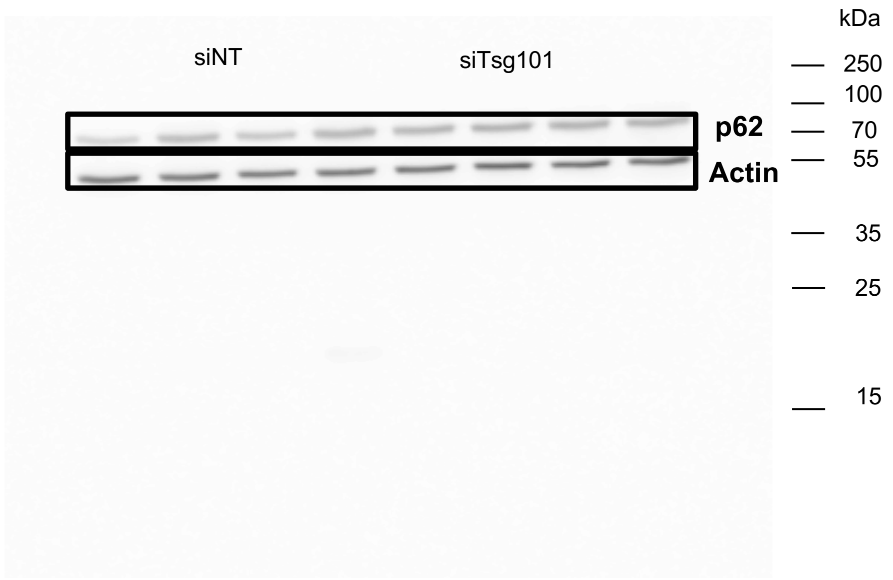

Supplementary Fig. 10

Uncropped data for Fig. 2b

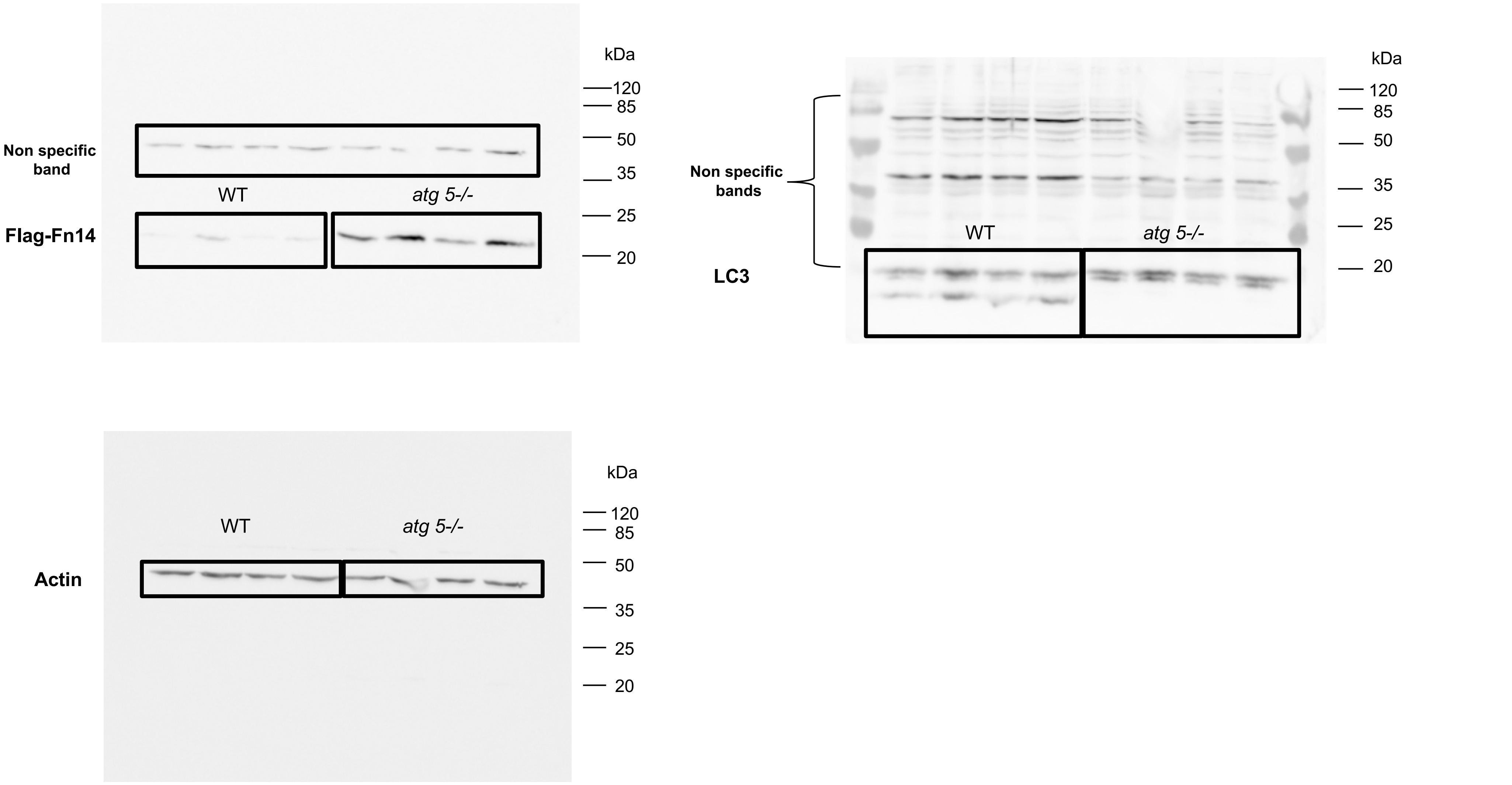

Supplementary Fig. 11

Uncropped data for Fig. 2c

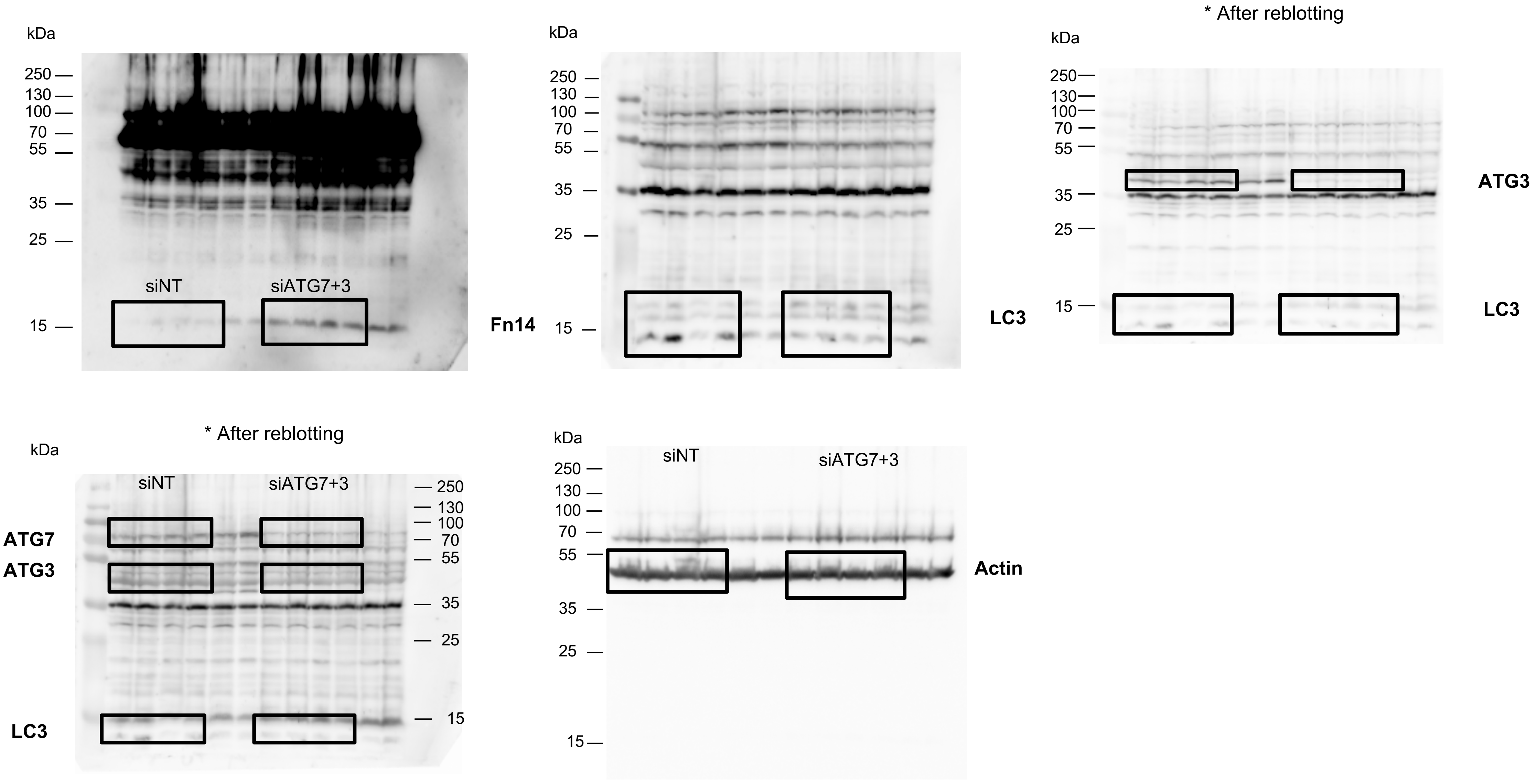

Supplementary Fig. 12

Uncropped data for Fig. 3d

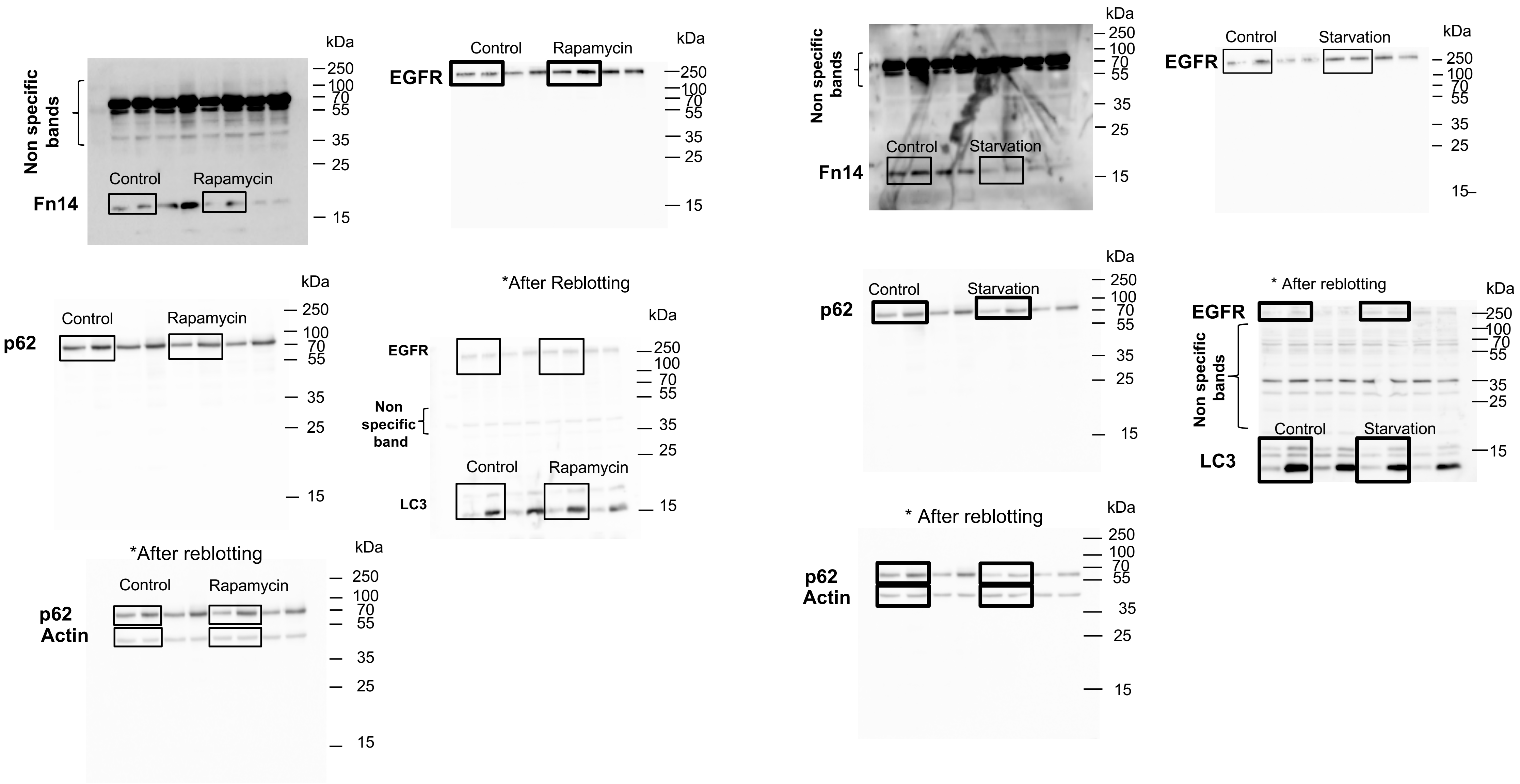

Supplementary Fig. 13

Uncropped data for Fig. 4b

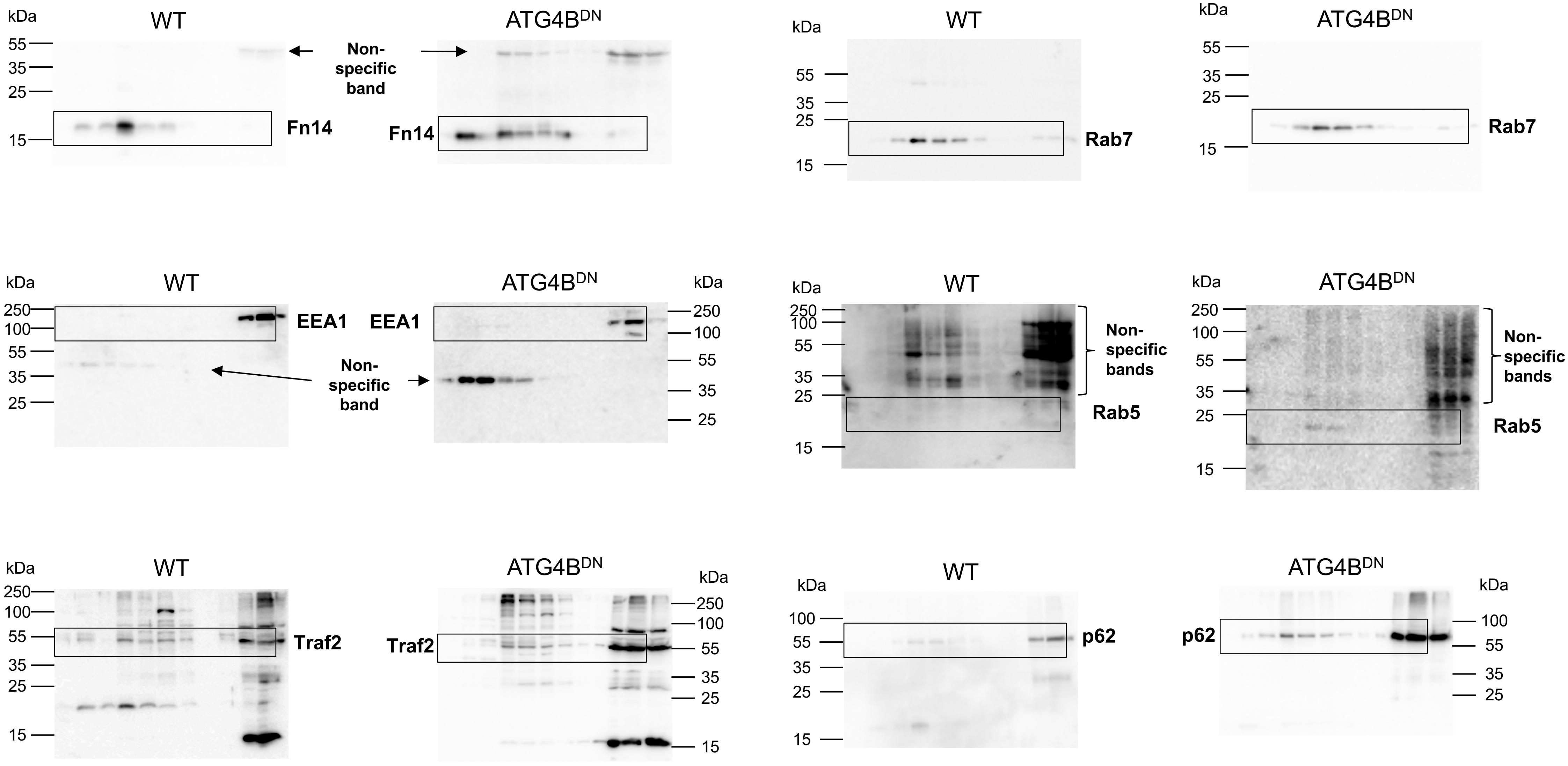

Supplementary Fig. 14

Uncropped data for Fig. 4d

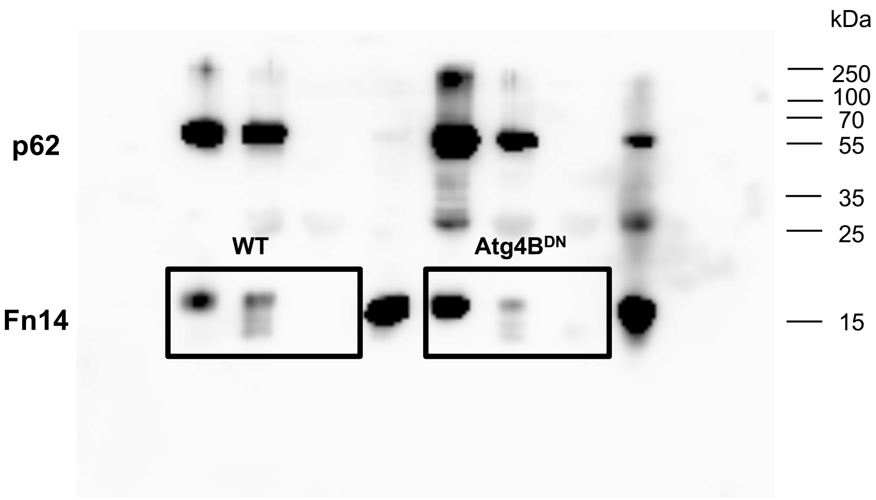

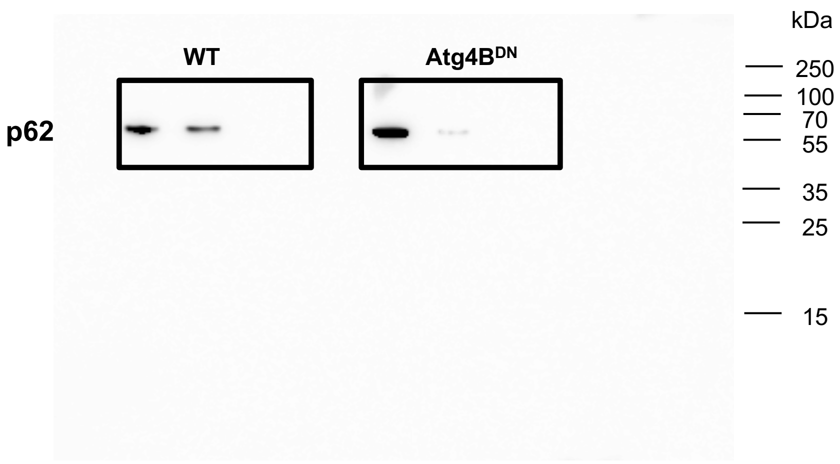

Supplementary Fig. 15

Uncropped data for Fig. 4e

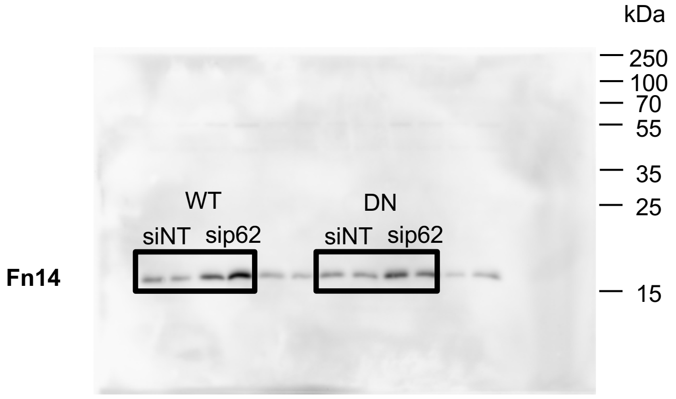

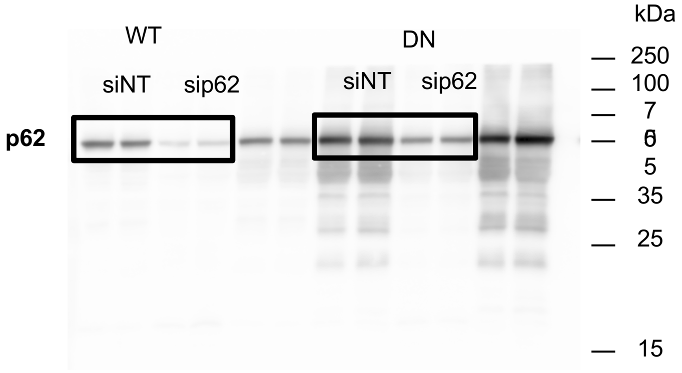

* After reblotting

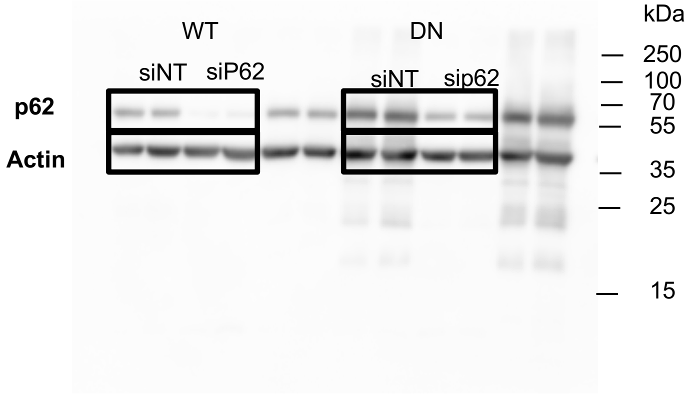

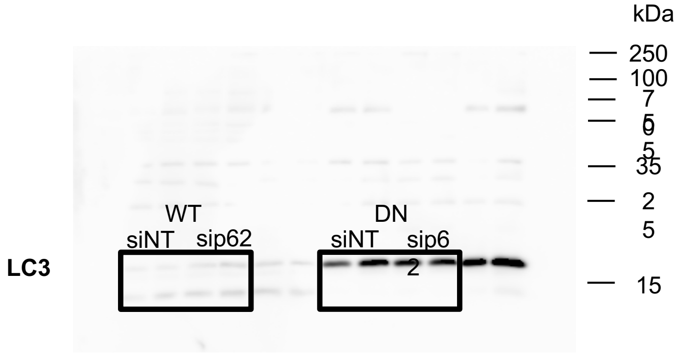

Supplement: Supplementary file 1 — Supplementary Information [file 41467_2018_6275_MOESM1_ESM.pdf]
